# Early intramedullary nailing of femoral shaft fracture on outcomes in patients with severe chest injury: A meta-analysis

**DOI:** 10.1038/srep30566

**Published:** 2016-07-26

**Authors:** Meng Jiang, Changli Li, Chengla Yi, Shaotao Tang

**Affiliations:** 1 Department of Pediatric Surgery, Union Hospital, Tongji Medical College, Huazhong University of Science and Technology, 1277 Jiefang Avenue, Wuhan 430022, Hubei Province, China; 2 Department of Geratology, Hubei Provincial Hospital of Integrated Chinese and Western medicine, 11 Lingjiaohu Avenue, Wuhan 430015, Hubei Province, China; 3 Department of Trauma Surgery, Tongji Hospital, Tongji Medical College, Huazhong University of Science and Technology, 1095 Jiefang Avenue, Wuhan 430022, Hubei Province, China

## Abstract

Early intramedullary nailing (IMN) within the first 24 hours for multiply injured patients with femoral fracture and concomitant severe chest injury is still controversial. This review aimed to investigate the association between early IMN and pulmonary complications in such patients. We searched the literature up to Jan 2016 in the main electronic databases (PubMed, Web of Science, Cochrane library databases) to identify eligible studies. Data were extracted and analyzed using a Mantel–Haenszel method with random-effects model to estimate pooled odds ratio (OR) and 95% confidence intervals (CIs). Seven retrospective cohort studies were identified eventually. The pooled estimates demonstrated that the application of early IMN did not significantly increase the risk of adult respiratory distress syndrome (ARDS) (OR, 0.65; 95% CI: 0.38–1.13), mortality (OR, 0.79; 95% CI: 0.43–1.47), pneumonia (OR, 0.92; 95% CI: 0.55–1.54), multiple organ failure (MOF) (OR, 0.87; 95% CI: 0.45–1.71) and pulmonary embolism (OR, 1.81; 95% CI: 0.28–11.83). In subgroup analysis according to the type of IMN (reamed or undreamed), we did not find any significant difference either. Our results indicated that early IMN of femoral shaft fracture was not associated with increased rates of pulmonary complications in severe chest-injured patients.

Femoral fracture combined with severe chest injury has become increasing common in orthopedic practice due to high-energy trauma. It was used to be treated with skeletal traction until patients were considered stable enough to undergo surgery for internal fixation^1^. However, during this procedure patients often developed adult respiratory distress syndrome (ARDS), infection, pneumonia, or even died, especially for those with a high Injury Severity Score (ISS)^2^,^3^. Several studies have explored how to decrease these severe complications and found that early surgical stabilization of the bone fracture could efficiently reduce the mortality and morbidity of multiply injured patients^4^,^5^,^6^,^7^.

In a recent study that comparing the efficient of different treatment options for femoral shaft fractures, the authors stated that femoral nailing was associated with the lowest complication rates compared with plating strategies and external fixation^8^. Actually, as a kind of biological internal fixation, intramedullary nailing (IMN) has been recommended as the golden standard in dealing with femoral shaft fractures^9^. However, for those multiply injured patients that have femoral fracture combined with severe chest injury, the use and timing of IMN is still controversial. In some investigator’s opinion the increased intramedullary pressure caused by IMN could release bone marrow and fat into the venous blood system, which may dramatically raise the incidence of ARDS and multiple organ failure (MOF)^10^. On the contrary, Carlson *et al*.^11^ reported that reamed intramedullary femoral fixation did not increase the pulmonary complication in severe chest injured patients. Thus, we conducted a meta-analysis to assess the safety of early IMN in patients with femoral shaft fracture and concurrent severe chest injury.

## Results

**Figure 1:**
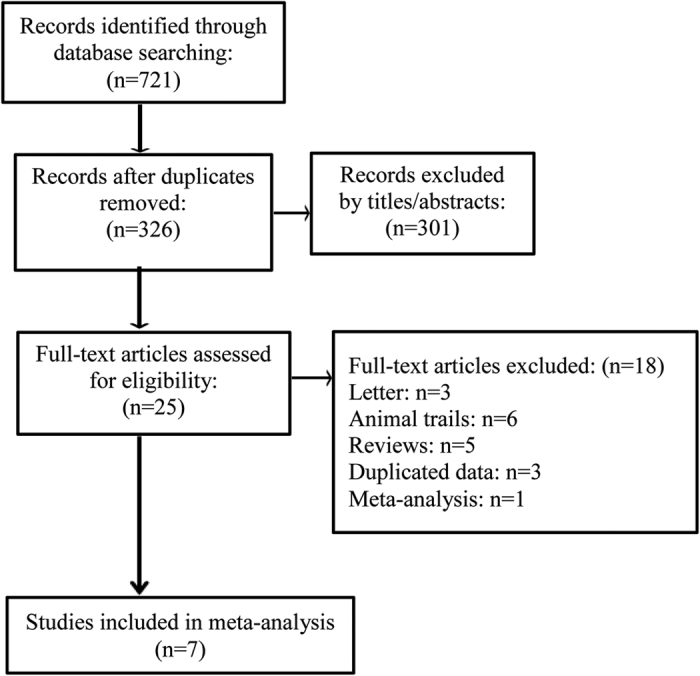
Flow chart of publication selection procedure.

We identified 721 potentially relevant citations according to our search strategy. After detailed screening and examination, 7 eligible studies were included in the final meta-analysis^11^,^12^,^13^,^14^,^15^,^16^,^17^. The selection process is shown in Fig. 1. All the 7 articles were retrospective cohort study. Across these studies, 1170 cases were included, with 277 patients underwent an early IMN stabilization.

Table 1 shows a summary of the characteristics of all studies included in our meta-analysis. The scores of the quality assessment are shown in Table 2. The kappa value in article selection and quality assessment were 0.89 and 0.84, respectively, both of which showed satisfactory agreement.

### Major Outcome: ARDS

**Figure 2:**
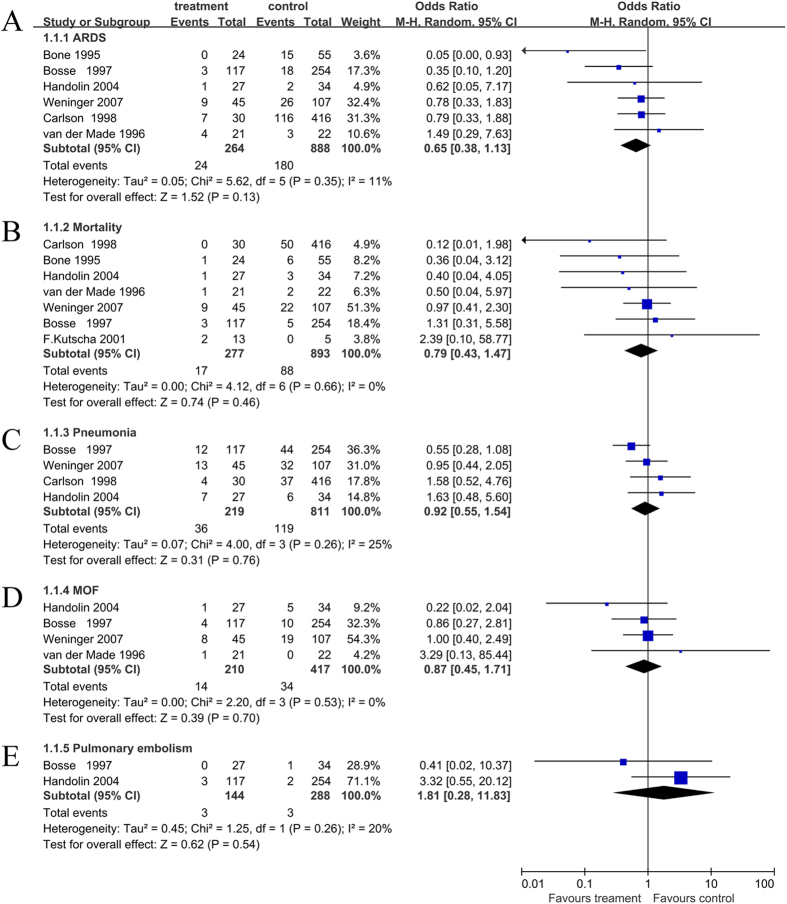
Forest plot for association between early IMN and clinical outcomes. (**A**) ARDS; (**B**) Mortality; (**C**) Pneumonia; (**D**) MOF; (**E**) pulmonary embolism.

**Figure 3:**
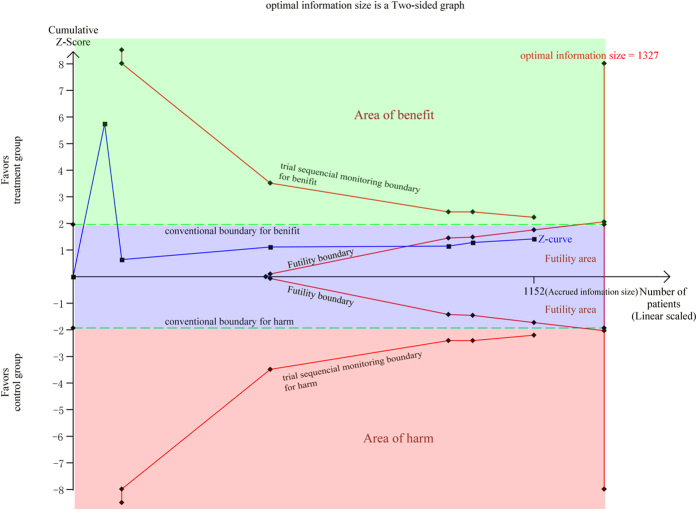
Trial sequential analysis of 6 trials for the association between early IMN and ARDS. The trial sequential analysis illustrating that the cumulative Z-curve crossed the Futility boundary and entered the Futility area, establishing sufficient and firm evidence that early IMN was not associated with higher risk of ARDS. The required optimal information size of 1,327 patients was calculated using α = 0.05 (two sided), β = 0.20 and a 25% control event rate (the rate of ARDS in the control group).

Six studies (1152 patients) have reported the incidence of ARDS between the two cohorts of patients^11^,^12^,^13^,^17^. These studies included 1,152 patients (sample size range, 18–371). The meta-analysis of the six studies showed no different incidence of ARDS among patients with and without early IMN stabilization (OR, 0.65; 95% CI: 0.38–1.13; I^2^ = 11%) (Fig. 2A). The trial sequential analysis for ARDS showed that the cumulative Z-curve crossed the Futility boundary and it entered into the Futility area, which means we can get sufficient and conclusive evidence that IMN was not involved with higher rate of ARDS (Fig. 3).

### Mortality

All included trials (1170 patients) reported the related mortality data^11^,^12^,^13^,^14^,^15^,^16^,^17^. Pooled analysis revealed that early IMN did not significantly increase the mortality in the multiply injured patients with femoral fracture and concomitant severe chest injury (OR, 0.79; 95% CI: 0.43–1.47; I^2^ = 0%) (Fig. 2B).

### Pneumonia

Four studies (1030 patients) reported the incidence of pneumonia between the two cohorts of patients^11^,^12^,^13^,^15^. The pooled results indicated no significant difference between the two groups (OR, 0.92; 95% CI: 0.55–1.54; I^2^ = 25%) (Fig. 2C).

### MOF

Four studies (627 patients) assessed the MOF rate in a total of 627 patients^12^,^13^,^15^,^16^. There were 210 patients who were treated by early IMN, in contrast to 253 patients who did not receive the surgery. Statistical analysis showed no difference between these patients (OR, 0.87; 95% CI: 0.45–1.71; I^2^ = 0%) (Fig. 2D).

### Pulmonary embolism

Pulmonary embolism was examined in two studies (432 patients)^13^,^15^. By pooling the studies, we did not find a markedly increased incidence of pulmonary embolism in the treatment group (OR, 1.81; 95% CI: 0.28–11.83); I^2^ = 20%) (Fig. 2E).

### Subgroup analysis and publication bias

**Figure 4:**
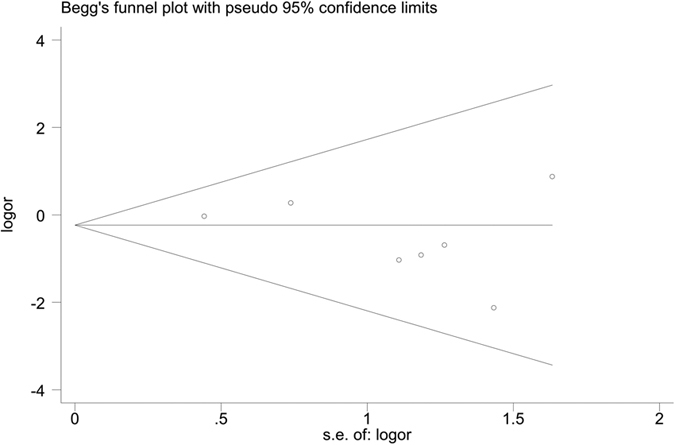
Begg’s funnel plot did not identify substantial asymmetry in the meta-analysis of total 7 studies.

In the subgroup analysis according to the type of IMN, we did not find statistical difference between outcomes of the treatment group and control group (Table 3). In addition, Begg’s funnel plot did not identify substantial asymmetry in the meta-analysis of total 7 studies (Fig. 4). The Begg’s rank correlation test and Egger’s linear regression test also showed no evidence of publication bias (Begg’s rank correlation test, P = 0.76; Egger’s linear regression test, P = 0.25).

## Discussion

Our meta-analysis identified seven retrospective cohort studies that investigating the safety of early IMN in patients with femoral fracture and coexisting severe chest injury. The systematic review did not demonstrate any additional respiratory complications and deaths caused by this treatment strategy. On the contrary, early stabilization of femoral fracture with intramedullary nail in these patients shows a trend (statistically insignificant) toward lower risk of ARDS.

IMN fixation is a minimally invasive and effective way to promote femoral fracture healing. It has become the structural method for most patients with femoral fractures^9^. Nevertheless, the surgical procedure can also release bone marrow and fat into the venous blood system and may exacerbate pulmonary function for multiply injured patients with severe chest injury. Concerns have been expressed about the potential adverse effect and many trauma surgeons have advocated the alternative of external fixation or skeletal traction in this context^10^,^18^. Pape *et al*.^19^ reported that primary IMN for femoral fracture could contribute to additional respiratory damage and may trigger ARDS in the presence of chest injury. In this study, patients with femoral fracture (ISS>18) treated by early IMN were grouped according to whether they have severe chest trauma. The results showed higher incidence of ARDS and mortality in the group who have severe chest injury. Basing on the data the authors concluded that it was the early IMN that account for the pulmonary dysfunction. However, the conclusion may provoke people to question that maybe it’s the presence of severe chest injury rather than IMN that lead to the worse outcomes.

Due to the abovementioned problem, many better designed studies were initiated to resolve this debate. Shortly afterward, Bone and colleagues^17^ performed a retrospective study to address this problem. In their study, the participants were divided into three groups: patients with femoral fractures and severe chest trauma undergoing early reamed IMN (group1), the similar injured patients who were stabilized with plate fixation (group2) and the patients with only severe chest injury (group3). The investigators found that there was a 33% ARDS rate in group2, a 27% rate in group3, and a 0% rate in group1. This study indicated that in the presence of severe chest trauma, early IMN will not increase the mortality and morbidity. This was confirmed by followed studies^15^,^16^.

In a latter research Pape and colleagues^20^ conducted a series of investigations including retrospective review, animal experiment, and prospective clinical study to explore the influence of chest injury and IMN on the incidence of ARDS in multiple-trauma patients. They found that unreamed early IMN was safe in patients with severe lung contusion. In an animal study in sheep, Gray *et al*.^21^ found that IMN indeed resulted in a significantly high initial pulmonary embolic load, however, there was no detectable effect on coagulation, pulmonary inflammation or animal mortality over the first 24 hours after injury. The pathophysiologic changes induced by IMN was not clinically significant to cause symptoms. This is consistent with the view of Bone *et al*.^22^, who deemed that the pulmonary complications seem to be secondary to the chest injury itself, but not the method of treatment for the femoral fracture fixation. By pooling all the illegible publications, our meta-analysis has contributed to the growing evidence that primary intramedullary femoral nailing did not cause additional pulmonary damage in the presence of severe chest injury.

Compared with unreamed IMN, the reamed type is associated with increased liberation of growth factors, and this is essential for fracture healing^23^. Despite four studies^11^,^15^,^16^,^17^ in our meta-analysis have confirmed the safety of reamed IMN in treating patients with severe chest injury, and subgroup analysis did not show any increased incidence of ARDS and mortality, the potential adverse effects from high intramedullary pressure during reaming procedure still warrants close attention. Streubel *et al*.^24^ had reported an application of reamer irrigator aspirator to reduce the pulmonary complications resulted from the reaming of the intramedullary cavity. Besides, in an animal study, Smith *et al*.^25^ developed an alternative approach that used an intramedullary suction system with multiple evacuation ports combined with a computerized monitoring system to control intramedullary pressure and prevent secondary fat embolization resulted from IMN. Both of these techniques can effectively reduce the potential risks during the reaming procedure.

It is important to emphasize that immediate and definitive fixation of fractures may not be beneficial for patients who are hemodynamically unstable or hypothermic, have poor oxygenation, or have coagulation abnormalities^26^. For these patients a “damage control orthopedic surgery” strategy has been advocated and is now widely accepted^27^. As long as no general contraindications for anesthesia and surgery present, early IMN should be initiated^9^.

The limitations of our meta-analysis should be acknowledged. First, in four publications^11^,^14^,^16^,^17^, the sex distribution were not reported. Thus a potential imbalance in sex between the groups may exist. Second, a major limitation of this literature is the lack of RCTs. Given that all included studies used retrospective designs, some of the outcomes (e.g. multi-organ failure) are “soft” and might be subject to coding differences. Third, some unmeasured confounding is still possible, particularly as all measures trended towards better outcomes in the early IMN group. This difference may be explained as sicker patients underwent delayed fixation because of concerns about exacerbating their chest injuries. Finally, although there were 1,170 patients (277 in the intervention group), some outcomes were very rare, e.g. only one pulmonary embolus recorded across the whole cohort. The confidence intervals for all outcomes were wide and it is possible that significant differences would have become apparent had more patients been available for analysis. Thus more RCTs would be required to evaluate these relationships and the part of our results should be explained with caution.

Taken together, our meta-analysis revealed that early intramedullary femoral nailing in multiply injured patients with severe chest injury is a safe treatment strategy, and it is not associated with higher risk of deterioration of lung function and mortality.

### Patients and Methods

This meta-analysis was performed and reported in line with the PRISMA and MOOSE guidelines^28^,^29^.

### Search strategy

We performed the systematic literature searches through PubMed, Web of Science and Cochrane library databases (Last search was updated on Jan 2016). Additionally, the reference lists of all identified publications and relevant reviews were searched manually for some other potential studies. The search strategy included the key words: (‘thoracic injury’ or ‘chest injury’ or ‘thoracic trauma’ or ‘chest trauma’) and (“femoral fracture” or “femur fracture”). No other restrictions such as language or primary objectives were inserted into the search strategy. The detailed search procedure is shown in the Fig. 1.

### Study Selection

Our inclusion criteria for choosing the eligible studies were listed as follows:

1. Population: multiply injured patients with the combination of a diaphyseal femoral fracture and severe chest trauma (Abbreviated Injury Scale [AIS] score ≥3), and the Injury Severity Score (ISS) ≥18.
2. Intervention: reamed or unreamed intramedullary fixation that performed less than 24 hours after injured.
3. Comparator: severe chest trauma without any lower extremity fracture.
4. Outcomes: the major outcome was acute respiratory distress syndrome (ARDS). Mortality, pneumonia, multiple organ failure (MOF) and pulmonary embolism were measured as the secondary outcomes.
5. Study design: randomized controlled trial, case-control study, prospective or retrospective cohort study.

### Data collection

Potential articles were assessed by screening titles or abstracts at first, followed by full-text review. Two investigators independently reviewed the identified full manuscripts for eligibility. The relevant data extracted from these articles included the name of the first author, country, severity of illness, study design, number of patients in each cohort, mean age, gender ratio, outcomes and the type of IMN. Any discrepancy was resolved by discussion or determined by a third author. Authors of the included studies were contacted if additional details were needed.

Descriptive case reports and reviews without comparative data were excluded. In the case of different articles related to the same patient population, only the reports with the longest time of follow-up and most number of cases were included.

As to studies included in this review compared early IMN fixation with a range of different control groups (ranging from lower limb non-femoral fractures to delayed treatment to plate fixation), we extracted the information from the original data of these studies to rebuild the intervention and comparator group.

### Study quality assessment

We assessed the quality of the included studies with a checklist adapted from Duckitt *et al*.^30^ and Taggart *et al*.^31^, which was based on the Newcastle Ottawa scale (NOS)^32^. The checklist contained 5 aspects: patient selection, comparability of groups at baseline, how the diagnosis of related outcomes were made, and group size as well as study design. Details of each item were outlined in Table 4. Studies without score in any item were excluded.

### Statistical analysis

The Cochrane Rev-Man software version 5.3 (Nordic Cochrane Center, Copenhagen, Denmark) and STATA 11.0 software (Stata Corporation, College Station, TX) were used for the statistical analysis. To measure the agreement between 2 investigators in study selection and quality assessment, a kappa statistic was performed. Heterogeneity among the included studies was assessed by I^2^ statistic using the heterogeneity Q statistic test. The heterogeneity was classified into low (I^2^ ≤ 25%), moderate (25% <I^2^ ≤ 50%), and high (I^2^ > 50%). The pooled effects of ARDS, mortality, pneumonia, MOF and pulmonary embolism syndrome were evaluated as odds ratio (OR) with 95% confidence intervals (CIs). In the quantitative synthesis, Mantel–Haenszel method with random-effects model was selected in consideration of the inherent clinical heterogeneity^33^. Subgroup analysis was conducted according to the type of surgical procedure (reamed or unreamed) to evaluate its influence on the outcomes. The funnel plot was performed by Begg’s rank correlation test to assess potential publication bias^34^. We also carried out the Egger’s linear regression test to assess the publication bias^35^. Any values of P < 0.05 was considered statistically significant.

### Trial Sequential Analysis

In a single randomized trial, monitoring boundaries are always used to decide whether it could be terminated early because the P value was sufficiently small to get a definitive conclusion. That means, we must calculate the sample size to ensure sufficient number of events and patients be included to allow for reliable statistical inference^36^. A similar “sample size” (optimal information size) is also essential for a meta-analysis, since we need to know if a meta-analysis at a given time could provide conclusive evidence of efficacy of a specific intervention^37^. So we did a trial sequential analysis to calculate the information size and monitoring boundaries to evaluate our meta-analysis^38^. Based on the results of the trial sequential analysis, we may be able to know whether further clinic trials should be performed and thus avoid spending resources on unnecessary further investigations.

For our trial sequential analysis of ARDS, we estimated the optimal information size using α = 0.05 (two sided), β = 0.20 and a 25% control event rate (the rate of ARDS in the control group). The software TSA version 0.9 beta (http://www.ctu.dk/tsa) was applied for this analysis.

## Additional Information

**How to cite this article**: Jiang, M. *et al*. Early intramedullary nailing of femoral shaft fracture on outcomes in patients with severe chest injury: A meta-analysis. *Sci. Rep.* **6**, 30566; doi: 10.1038/srep30566 (2016).

## Figures and Tables

**Table 1:** Characteristics of seven studies included in this meta-analysis.

study		Country	Severity of illness	Study design	No. Patients		Mean Age, y		Male Sex, %		outcomes	Type of IMN
					SC	CC	SC	CC	SC	CC		
Weninger^12^	2007	Austria	Need for mechanical ventilation; Thoracic AIS≥3; ISS≥18	Retrospective cohort	45	107	33.4	32.2	69	67	Pneumonia, ARDS, MOF, Mortality	Unreamed
Handolin^13^	2004	Finland	Unilateral or bilateral pulmonary contusion; Need for mechanical ventilation; Thoracic AIS≥3	Retrospective cohort	27	34	39	38	70	62	Pneumonia, ARDS, MOF, Mortality, Pulmonary embolism	NR
F.Kutscha^14^	2001	Germany	Thoracic AIS≥3; ISS≥18	Retrospective cohort	13	5	28.6	38.2	NR	NR	Mortality	Unreamed
Bone^17^	1995	America	Thoracic AIS≥3 (hemopneumothorax, multiple rib fractures, or pulmonary contusion); ISS≥18	Retrospective cohort	24	55	36.6	47.3	NR	NR	ARDS, Mortality	Reamed
Carlson^11^	1998	America	Thoracic AIS≥3	Retrospective cohort	30	416	29	39	NR	NR	Pneumonia, ARDS Mortality	Reamed
Bosse^15^	1997	America	ISS≥18; Thoracic AIS≥3	Retrospective cohort	117	254	28	29	79	69	Pneumonia, ARDS, MOF, Mortality, Pulmonary, embolism	Reamed
van der Made^16^	1996	Netherlands	ISS>25; no mortality<24 h; rHTI≥3	Retrospective cohort	21	22	36.1	36	NR	NR	ARDS, MOF, Mortality	Reamed

ISS: Injury Severity Score; SC: study cohort; CC: control cohort; NR: not reported; IMN: intramedullary nailing; rHTI: respiratory of hospital trauma index; ARDS: Acute Respiratory Distress Syndrome; MOF: Multiple Organ Failure; AIS: Abbreviated Injury Scale.

**Table 2:** Quality scores of included studies.

Included study	Selection	Comparability	Outcome	Size	Cohort design
Weninger^12^	1	2	2	1	1
Handolin^13^	1	2	2	1	1
F.Kutscha^14^	1	1	1	1	1
Bone^17^	1	0	2	1	1
Carlson^11^	1	2	2	1	1
Bosse^15^	1	2	2	2	1
van der Made^16^	1	2	2	1	1

**Table 3:** Subgroup analysis according to the type of IMN (by Mantel–Haenszel method with randomized effects model).

Stratification	No. of patients (studies)	No. of events/No. in group		OR (95% CI)	P value for heterogeneity	I^2^, %
		Treatment	Control			
Mortality						
Reamed IMN	939(4)	5/192	63/747	0.62(0.22–1.70)	0.4	0
Unreamed IMN	170(2)	11/58	22/112	1.03(0.44–2.38)	0.59	0
ARDS						
Reamed IMN	939(4)	14/192	152/747	0.55(0.21–1.44)	0.14	45
Unreamed IMN	152(1)	9/45	26/107	0.78(0.33–1.83)	0.57	—
Pneumonia						
Reamed IMN	817(2)	16/147	81/670	0.85(0.30–2.36)	0.11	61
Unreamed IMN	152(1)	13/45	32/107	0.95(0.44–2.05)	0.9	—
MOF						
Reamed IMN	414(2)	5/138	10/276	1.01(0.33–3.06)	0.45	0
Unreamed IMN	152(1)	8/45	19/107	1.00(0.40–2.49)	1	—
Pulmonary						
embolism						
Reamed IMN	61(1)	0/27	1/34	0.41(0.02–10.37)	0.59	—
Unreamed IMN	—	—	—	—	—	—

ARDS: Acute Respiratory Distress Syndrome; MOF: Multiple Organ Failure; IMN: intramedullary nailing.

**Table 4:** Quality assessment of non-randomized studies.

**Participant selection**
Selected cohort was representative of the multiply injured patients with femoral shaft fracture and concomitant severe chest injury (1)
Cohort was a selected group but the selection was not described (0)
**Comparability of groups**
No differences between the groups explicitly reported (especially in terms of age, gender, ISS score, thoracic AIS score and pre-existing disease) unless it was one of these variables that was under investigation, or such differences were adjusted for (2)
Differences between groups were not recorded (1)
Groups differed (0)
**Outcomes**
Referenced definition of clinic outcomes including ARDS, mortality, pneumonia, MOF and fat embolism syndrome (1)
Outcomes were not defined (0)
**Group size**
>100 participants in each group (2)
<100 participants in each group (1)
**Cohort design**
Prospective design (2)
Retrospective design (1)

## References

[b1] Bradford D. S., Foster R. R. & Nossel H. L. Coagulation alterations, hypoxemia, and fat embolism in fracture patients. J Trauma 10, 307–321 (1970). 5435842 10.1097/00005373-197004000-00004

[b2] Baker S. P., O’Neill B., Haddon W. J. & Long W. B. The injury severity score: a method for describing patients with multiple injuries and evaluating emergency care. J Trauma 14, 187–196 (1974). 4814394

[b3] Bone L. B., Johnson K. D., Weigelt J. & Scheinberg R. Early versus delayed stabilization of femoral fractures. A prospective randomized study. J. Bone Joint Surg Am 71, 336–340 (1989). 2925704

[b4] Goris R. J., Gimbrere J. S., van Niekerk J. L., Schoots F. J. & Booy L. H. Early osteosynthesis and prophylactic mechanical ventilation in the multitrauma patient. J Trauma 22, 895–903 (1982). 6754962 10.1097/00005373-198211000-00002

[b5] Johnson K. D., Cadambi A. & Seibert G. B. Incidence of adult respiratory distress syndrome in patients with multiple musculoskeletal injuries: effect of early operative stabilization of fractures. J Trauma 25, 375–384 (1985). 3999159 10.1097/00005373-198505000-00001

[b6] Meek R. N., Vivoda E. E. & Pirani S. Comparison of mortality of patients with multiple injuries according to type of fracture treatment–a retrospective age- and injury-matched series. Injury 17, 2–4 (1986). 3770879 10.1016/0020-1383(86)90003-3

[b7] Nahm N. J. & Vallier H. A. Timing of definitive treatment of femoral shaft fractures in patients with multiple injuries: A systematic review of randomized and nonrandomized trials. J. Trauma Acute Care 73, 1046–1063 (2012). 10.1097/TA.0b013e3182701ded 23117368

[b8] Ramseier L. E., Janicki J. A., Weir S. & Narayanan U. G. Femoral fractures in adolescents: a comparison of four methods of fixation. J. Bone Joint Surg Am 92, 1122–1129 (2010). 20439657 10.2106/JBJS.H.01735

[b9] Gansslen A., Gosling T., Hildebrand F., Pape H. C. & Oestern H. J. Femoral shaft fractures in adults: treatment options and controversies. Acta Chir Orthop Traumatol Cech 81, 108–117 (2014). 25105784

[b10] Schult M. F. U. & Pathophysiology F. S. E. B. of Fat Embolism after Intramedullary Reaming. J Trauma 29, 68–73 (2003).

[b11] Carlson D. W., Rodman G. J., Kaehr D., Hage J. & Misinski M. Femur fractures in chest-injured patients: is reaming contraindicated? J. Orthop Trauma 12, 164–168 (1998). 9553856 10.1097/00005131-199803000-00005

[b12] Weninger P., Figl M., Spitaler R., Mauritz W. & Hertz H. Early unreamed intramedullary nailing of femoral fractures is safe in patients with severe thoracic trauma. J Trauma 62, 692–696 (2007). 17414349 10.1097/01.ta.0000243203.38466.e0

[b13] Handolin L., Pajarinen J., Lassus J. & Tulikoura I. Early intramedullary nailing of lower extremity fracture and respiratory function in polytraumatized patients with a chest injury: a retrospective study of 61 patients. Acta Orthop Scand 75, 477–480 (2004). 15370594 10.1080/00016470410001277-1

[b14] Kutscha-Lissberg F., Hopf F. K., Kollig E. & Muhr G. How risky is early intramedullary nailing of femoral fractures in polytraumatized patients? Injury 32, 289–293 (2001). 11325364 10.1016/s0020-1383(00)00202-3

[b15] Bosse M. J. . Adult respiratory distress syndrome, pneumonia, and mortality following thoracic injury and a femoral fracture treated either with intramedullary nailing with reaming or with a plate. A comparative study. J. Bone Joint Surg Am 79, 799–809 (1997). 9199375 10.2106/00004623-199706000-00001

[b16] van der Made W. J., Smit E. J., van Luyt P. A. & van Vugt A. B. Intramedullary femoral osteosynthesis: an additional cause of ARDS in multiply injured patients? Injury 27, 391–393 (1996). 8881133 10.1016/0020-1383(96)00040-x

[b17] Bone L. B., Babikian G. & Stegemann P. M. Femoral canal reaming in the polytrauma patient with chest injury. A clinical perspective. Clin Orthop Relat Res 318, 91–94 (1995). 7671536

[b18] Aufmkolk M. . Effect of primary femoral plate osteosynthesis on the course of polytrauma patients with or without thoracic trauma. Unfallchirurg 101, 433–439 (1998). 9677841 10.1007/s001130050292

[b19] Pape H. C. . Primary intramedullary femur fixation in multiple trauma patients with associated lung contusion–a cause of posttraumatic ARDS? J Trauma 34, 540–547, 547–548 (1993). 10.1097/00005373-199304000-00010 8487339

[b20] Pape H. C., Regel G., Dwenger A., Sturm J. A. & Tscherne H. Influence of thoracic trauma and primary femoral intramedullary nailing on the incidence of ARDS in multiple trauma patients. Injury 24 Suppl 3, S82–S103 (1993). 8168882 10.1016/0020-1383(93)90012-u

[b21] Gray A. C. . The stress response to bilateral femoral fractures: a comparison of primary intramedullary nailing and external fixation. J. Orthop Trauma 23, 90–99 (2009). 19169099 10.1097/BOT.0b013e31819194c6

[b22] Bone L. B., Anders M. J. & Rohrbacher B. J. Treatment of femoral fractures in the multiply injured patient with thoracic injury. Clin Orthop Relat Res 347, 57–61 (1998). 9520875

[b23] Giannoudis P. V. . Growth factor release following femoral nailing. Bone 42, 751–757 (2008). 18243089 10.1016/j.bone.2007.12.219

[b24] Streubel P. N., Desai P. & Suk M. Comparison of RIA and conventional reamed nailing for treatment of femur shaft fractures. Injury 41 Suppl 2, S51–S56 (2010). 21144929 10.1016/S0020-1383(10)70010-3

[b25] Smith P. N. . Monitoring and controlling intramedullary pressure increase in long bone instrumentation: a study on sheep. J. Orthop Res 26, 1327–1333 (2008). 18464262 10.1002/jor.20564

[b26] Bone L. B. & Giannoudis P. Femoral shaft fracture fixation and chest injury after polytrauma. J. Bone Joint Surg Am 93, 311–317 (2011). 21266645 10.2106/JBJS.J.00334

[b27] Scalea T. M. . External fixation as a bridge to intramedullary nailing for patients with multiple injuries and with femur fractures: damage control orthopedics. J Trauma 48, 613–623 (2000). 10780592 10.1097/00005373-200004000-00006

[b28] Moher D., Liberati A., Tetzlaff J. & Altman D. G. Preferred reporting items for systematic reviews and meta-analyses: the PRISMA statement. BMJ 339, b2535 (2009). 19622551 10.1136/bmj.b2535 PMC2714657

[b29] Stroup D. F. . Meta-analysis of observational studies in epidemiology: a proposal for reporting. Meta-analysis Of Observational Studies in Epidemiology (MOOSE) group. JAMA 283, 2008–2012 (2000). 10789670 10.1001/jama.283.15.2008

[b30] Duckitt K. & Harrington D. Risk factors for pre-eclampsia at antenatal booking: systematic review of controlled studies. BMJ 330, 565 (2005). 15743856 10.1136/bmj.38380.674340.E0 PMC554027

[b31] Taggart D. P., D’Amico R. & Altman D. G. Effect of arterial revascularisation on survival: a systematic review of studies comparing bilateral and single internal mammary arteries. Lancet 358, 870–875 (2001). 11567701 10.1016/S0140-6736(01)06069-X

[b32] Wells G. A. . The Newcastle-Ottawa Scale (NOS) for assessing the quality of nonrandomized studies in meta-analyses. Ottawa Health Research Institute Web site (2016).

[b33] DerSimonian R. & Laird N. Meta-analysis in clinical trials. Control Clin Trials 7, 177–188 (1986). 3802833 10.1016/0197-2456(86)90046-2

[b34] Begg C. B. & Mazumdar M. Operating characteristics of a rank correlation test for publication bias. Biometrics 50, 1088–1101 (1994). 7786990

[b35] Egger M., Davey S. G., Schneider M. & Minder C. Bias in meta-analysis detected by a simple, graphical test. BMJ 315, 629–634 (1997). 9310563 10.1136/bmj.315.7109.629 PMC2127453

[b36] Moher D., Schulz K. F. & Altman D. G. The CONSORT statement: revised recommendations for improving the quality of reports of parallel-group randomised trials. Lancet 357, 1191–1194 (2001). 11323066

[b37] Pogue J. M. & Yusuf S. Cumulating evidence from randomized trials: utilizing sequential monitoring boundaries for cumulative meta-analysis. Control Clin Trials 18, 580–593, 661–666 (1997). 10.1016/s0197-2456(97)00051-2 9408720

[b38] Brok J., Thorlund K., Gluud C. & Wetterslev J. Trial sequential analysis reveals insufficient information size and potentially false positive results in many meta-analyses. J. Clin Epidemiol 61, 763–769 (2008). 18411040 10.1016/j.jclinepi.2007.10.007

